# Endogenous Endophthalmitis Caused by ST66-K2 Hypervirulent *Klebsiella pneumoniae*, United States

**DOI:** 10.3201/eid2708.210234

**Published:** 2021-08

**Authors:** Edwin Kamau, Paul R. Allyn, Omer E. Beaird, Kevin W. Ward, Nancy Kwan, Omai B. Garner, Shangxin Yang

**Affiliations:** University of California, Los Angeles, California, USA

**Keywords:** endogenous endophthalmitis, hypervirulent *Klebsiella pneumoniae*, *K. pneumoniae*, ST66-K2, bacteria, United States

## Abstract

We describe a case of endogenous endophthalmitis caused by sequence type 66-K2 hypervirulent *Klebsiella pneumoniae* in a diabetic patient with no travel history outside the United States. Genomic analysis showed the pathogen has remained highly conserved, retaining >98% genetic similarity to the original strain described in Indonesia in 1935.

Hypervirulent *Klebsiella pneumoniae* (hvKp) strains are mostly community-acquired and can cause invasive infections such as liver abscess with metastatic spread (*1*,*2*). The genetic determinants of hypervirulence are found on chromosomal mobile genetic elements, large plasmids, or both. The most common virulence determinants of hvKp include siderophore systems for iron acquisition, increased capsule production, K1 and K2 serotypes, and the colibactin toxin (*1*). In addition, these hvKp strains demonstrate hypermucoviscosity, as indicated by a positive string test, and are usually susceptible to antimicrobial drugs (*1*). However, multidrug‐resistant hypervirulent strains have emerged in Asia, a region to which hvKp is endemic (*1*,*3*). Kp52.145 (laboratory strain B5055), which belongs to sequence type (ST) 66, is one of the most virulent and widely studied K2 strains. The ST66-K2 sublineage contains virulence genes in its chromosome and 2 large plasmids (*4*,*5*). ST66-K2 was isolated in Indonesia in 1935; since then, cases have been reported in Australia in 2002 (caused by strain AJ210), Germany in 2017 (caused by strain 18-0005) and France in 2018 (caused by strain SB5881) (*6*–*8*).

The most common hvKp infection metastatic sites are the eyes, lungs, and central nervous system (*1*). Endogenous endophthalmitis (EE) caused by hvKp is associated with risk factors such as diabetes mellitus, Asian ancestry, and infection with the K1 serotype (*2*). Although the prevalence of hvKp is increasing in the United States and Europe (*1*,*2*,*9*), where EE has been documented in patients of Asian and non-Asian descent (*9*,*10*), these infections are not well-recognized. Ocular prognoses and clinical outcomes for EE are usually poor and exacerbated by late or missed diagnosis (*2*). We describe a case of EE caused by a hvKp strain of the ST66-K2 sublineage in the United States.

## The Study

A 30-year-old Caucasian man who had a history of poorly controlled type 1 diabetes mellitus and recreational use of methamphetamine and intravenous heroin sought treatment at the emergency department of a local hospital in California, USA, for progressive right eye and ear pain, which had lasted ≈1 week, and vision loss. Hospital staff noted substantial edema and tenderness of the right external auditory canal with otorrhea, along with suspected orbital cellulitis. Computed tomography scans revealed complete opacification of the right middle ear cavity and mastoid air cells, prominent thickening and hyperenhancement of the right posterolateral sclera, and a cystic and necrotic lesion in the left parotid region. He was prescribed vancomycin and cefepime and then transferred to Ronald Reagan UCLA Medical Center (Los Angeles, CA, USA) for ophthalmologic evaluation.  At admission, he had a perforated right tympanic membrane with external otitis media and mastoiditis, a left parotid abscess, and right endogenous endophthalmitis with subretinal abscess. A transthoracic echocardiogram showed no signs of valvular vegetations; an abdominal ultrasound showed no signs of hepatic lesions. Results of blood cultures were negative. Cultures from the parotid abscess and ear drainage grew hypermucoviscous *K. pneumoniae* (Appendix Figure 1) and methicillin-susceptible *Staphylococcus aureus*. The *K. pnuemoniae* isolate was susceptible to all drugs tested, including ampicillin (Appendix Table 1).

We prescribed intravitreal injections of vancomycin, ceftazidime, and voriconazole every other day in addition to intravenous ceftriaxone (2 g 2×/d), intravenous voriconazole (4 mg/kg 2×/d), and oral metronidazole (500 mg 3×/d) for ≈2 weeks. We conducted a pars plana vitrectomy to drain the subretinal abscess in the patient’s right eye. We sent the vitreous and aqueous samples for bacterial and fungal culturing, which returned negative results. After the surgery, the patient continued to take ceftriaxone, metronidazole, and voriconazole in addition to using eye drops containing prednisolone, ciloxan, and atropine. He was discharged 15 days after admission. Two weeks after discharge, he reported that his pain had resolved but his vision loss continued with minimal light perception.

We sequenced the isolate (UCLA353) using the Miseq platform (Illumina, https://www.illumina.com) with 2 × 250 bp protocol; long-read sequencing was conducted using MinION (Oxford Nanopore Technologies, https://nanoporetech.com) according to the manufacturer’s recommendations. The sequence files were submitted to the National Center for Biotechnology Information Sequence Read Archive (https://www.ncbi.nlm.nih.gov/sra) under BioProject accession no. PRJNA729785. We analyzed the sequences using the CLC Genomics Workbench (QIAGEN, https://www.qiagen.com) and Geneious Prime (Geneious, https://www.geneious.com). We identified multilocus sequence types and virulence factors using BIGSdb (https://bigsdb.pasteur.fr/klebsiella). In addition, we used the default settings of ResFinder to identify antimicrobial resistance genes, CSI Phylogeny to identify single-nucleotide polymorphisms (SNPs), and PlasmidFinder to identify plasmid replicons (Center for Genomic Epidemiology, https://cge.cbs.dtu.dk/services). Genomic analyses revealed UCLA353 to be closely related to Kp52.145 (GenBank accession no. FO834906) with 99.7% genomic coverage and 98.8% pairwise identity. UCLA353 and Kp52.145 had all identical chromosomal hypervirulent genes and genomic islands, including the K2 capsular gene cluster, colibactin gene, yersiniabactin gene on an ICE*Kp*10 mobile genetic element, and the recently described phospholipase D family protein gene (Figure) (*11*). In addition, UCLA353 carried 2 plasmids (with lengths of 95,157 bp and 164,217 bp) nearly identical to those present in the SB5881 isolate documented in 2018 in France (GenBank accession nos. LR792629 and LR792630), with 100% genomic coverage and 99.9% pairwise identity (Appendix Figure 2). The 95-kb plasmid I in UCLA353 was also nearly identical to the Kp52.145 plasmid I (GenBank accession no. FO834904); the 164-kb plasmid II shared 100% genome coverage with Kp52.145 plasmid II (FO834905) but had a 39-kb sequence insertion previously described in SB5881 (*8*) (Appendix Figure 2). SNP analysis of the chromosomal sequences of UCLA353 and the other 4 ST66-K2 strains revealed that UCLA353 was genetically distinct, with 775 SNPs compared with Kp52.145 and 785–802 compared with AJ210, 18-0005, and SB5881 (Table ). UCLA353 did not carry any resistance genes. Similar to other ST66-K2 strains, UCLA353 did not have the *bla*_SHV_ gene, and was therefore susceptible to β-lactams including ampicillin (Appendix Table 1). Further analysis showed all the ST66-K2 strains carried highly similar virulence factors (Appendix Table 2).

**Figure:**
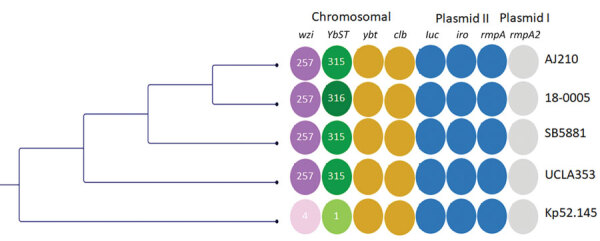
Comparative genetic analysis of sequence type 66-K2 hypervirulent *Klebsiella pneumoniae* isolate (UCLA353) from a 30-year-old man in California, USA, who had endogenous endophthalmitis and 4 other isolates: AJ210 (Australia, 2002 [*6*]), 18-0005 (Germany, 2017 [*7*]), SB5881 (France, 2018 [*8*]), and Kp52.145 (Indonesia, 1935 [[*11*]). Maximum-likelihood tree based on single-nucleotide polymorphisms and not drawn to scale. Colors indicate different loci; shades indicate different alleles. Colored columns show the capsular sequence type of the *wzi* gene, which codes for the outer membrane protein WZI; YbST; the chromosomal virulence loci y*ybt* and *clb*; the plasmid II–associated virulence loci *iuc*, *iro*, and *rmpA*; and the plasmid I–associated virulence locus *rmpA2*. AJ210, 18-0005, SB5881 and UCLA353 share the *wzi* 257 allele (dark purple). AJ210, SB5881 and UCLA353 share the YbST 315 allele, whereas 18-0005 has the YbST 316 allele (dark green). The *wzi* and YbST alleles for strain Kp52.145 are shown in lighter colors. *clb*, colibactin; *iro*, salmochelin; *iuc*, aerobactin; *rmpA*, regulator of mucoid phenotype; YbST, yersiniabactin sequence type; *ybt*, yersiniabactin.

**Table:** Single-nucleotide polymorphism matrix of 5 ST66-K2 hypervirulent *Klebsiella pneumoniae* strains

Strain (country, year [reference])	18-0005	AJ210	Kp52.145	SB5881	UCLA353
18-0005 (Germany, 2017 [*7*])	0	65	219	56	796
AJ210 (Australia, 2002 [*6*])	65	0	208	71	785
Kp52.145 (Indonesia, 1935 [*11*])	219	208	0	225	775
SB5881 (France, 2018 [*8*])	56	71	225	0	802
UCLA353 (United States, 2020, this study)*	796	785	775	802	0

*Isolate from a 30-year-old man in California who had endogenous endophthalmitis.

## Conclusions

Ocular prognoses and clinical outcomes for EE are usually poor, often entailing partial or complete vision loss, enucleation or evisceration, or death (*2*). Late or missed diagnosis delays the initiation of specialized ocular therapy (e.g., intravitreal or source control) and can worsen outcomes. Early treatment is crucial to preserving full or partial vision (*1*–*3*,*10*). A pooled analysis of clinical studies revealed that most (83.2%) EE infections caused by hvKp were detected >24 hours after admission (*2*). These data indicate that patients at high risk for EE, especially those with underlying conditions such as diabetes mellitus or *K. pneumoniae*–associated pyogenic liver abscess, should be monitored closely for EE even when it is not initially apparent. Detection of K1 or K2 capsular serotypes, hypermucoviscous phenotype, and ampicillin susceptibility might suggest disseminated EE caused by hvKp. Although bacteremia is usually a prerequisite for metastatic dissemination, it may not always be detectable (*1*,*2*).

This infection probably began as otitis externa complicated by otitis media caused by perforated tympanic membrane and otomastoiditis, conditions that subsequently spread to the sinuses and right orbit. In a similar scenario, strain SB5881 also caused invasive infection including acute otitis media in a patient with type 1 diabetes mellitus and chronic alcoholism (*8*). Despite its emergence in or before 1935, ST66-K2 hvKp infections were not reported until 2002, probably because of the limited availability of high-resolution genomic sequencing tools in the 20th century (*8*,*11*). Thus, the prevalence of ST66-K2 hvKp might be largely underestimated.

In summary, we describe a case of EE caused by ST66-K2 hvKp in a Caucasian diabetic man with no travel history outside the United States. This lineage has remained highly conserved, preserving all of its virulence factors and >98% of its genome. Clinicians should be aware of the threat and challenges of EE caused hvKp infections.

Appendix Additional data on endogenous endophthalmitis caused by sequence type 66-K2 hypervirulent *Klebsiella pneumoniae*, United States.
