# Supplementary material for: Endogenous Endophthalmitis Caused by ST66-K2 Hypervirulent Klebsiella pneumoniae, United States
Source: Emerg Infect Dis. 2021 Aug;27(8):2215–8. doi: 10.3201/eid2708.210234 (PMC8314818; doi:10.3201/eid2708.210234)
Supplement: Appendix — Additional data on endogenous endophthalmitis caused by sequence type 66-K2 hypervirulent Klebsiella pneumoniae, United States. [file 21-0234-Techapp-s1.pdf]

Article DOI: https://doi.org/10.3201/eid2708.210234

Endogenous Endophthalmitis Caused by ST66-K2 Hypervirulent *Klebsiella pneumoniae*, United States

Appendix

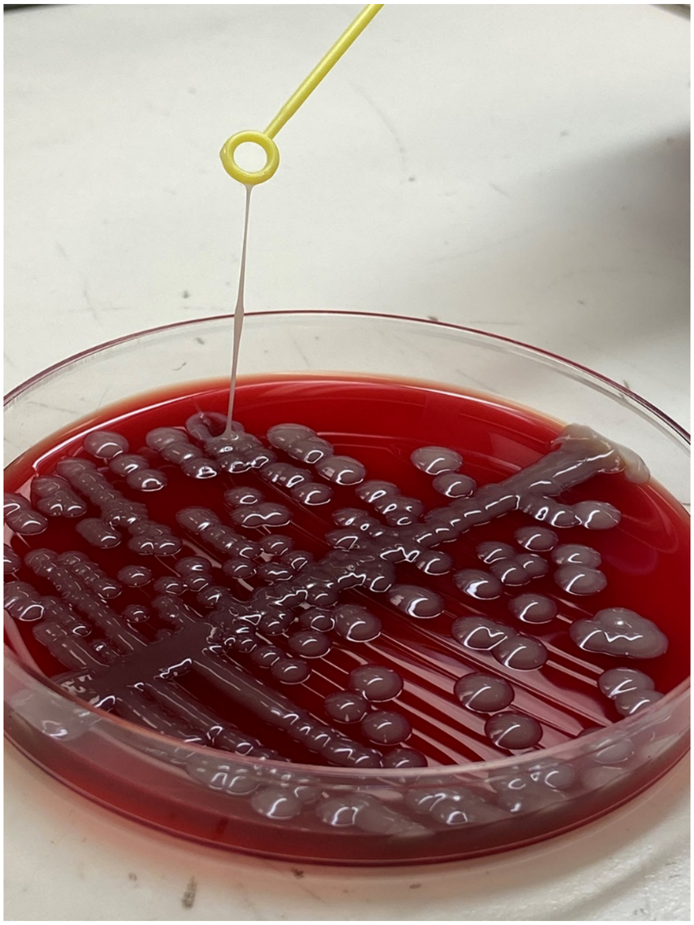

Appendix Figure 1. Positive string test for hypermucoviscuous *Klebsiella pneumoniae* cultures grown from left parotid abscess and right ear drainage samples of a patient with endogenous endophthalmitis, United States.

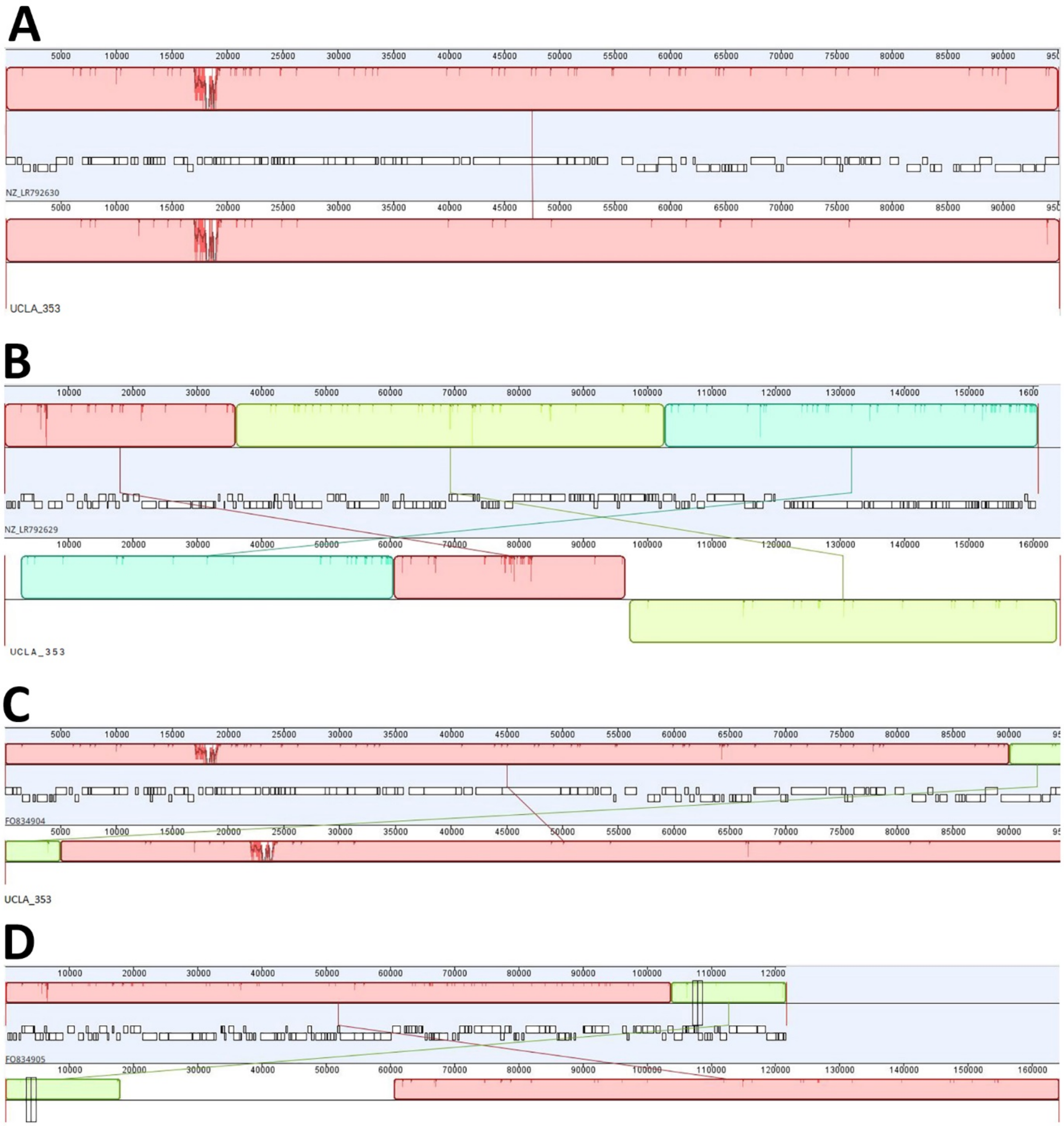

Appendix Figure 2. Whole-genome alignment of *Klebsiella pneumoniae* plasmids. Values indicate base pairs. Corresponding colors indicate nearly identical sequences. A) UCLA353 plasmid I and SB5881 plasmid I (GenBank accession no. LR792630). B) UCLA353 plasmid II and SB5881 plasmid II (GenBank accession no. LR792629). C) UCLA353 plasmid I and Kp52.145 plasmid I (GenBank accession no. FO834904). D) UCLA353 plasmid II and Kp52.145 plasmid II (GenBank accession no. FO834905). Images generated by Geneious Prime (Geneious, https://www.geneious.com).

Appendix Table 1. Antimicrobial susceptibility profile of *Klebsiella pneumoniae* strain UCLA353

Antimicrobial drug	Minimal inhibitory concentration, μg/mL	Interpretation
Piperacillin/tazobactam	≤8	Susceptible
Cefazolin	2	Susceptible
Ceftriaxone	≤1	Susceptible
Ceftazidime	≤0.5	Susceptible
Ceftolozane/tazobactam	≤0.5	Susceptible
Cefepime	≤0.5	Susceptible
Imipenem	≤0.25	Susceptible
Meropenem	≤0.25	Susceptible
Gentamicin	≤1	Susceptible
Tobramycin	≤1	Susceptible
Amikacin	≤4	Susceptible
Ceftazidime/avibactam	≤2	Susceptible
Ertapenem	≤0.25	Susceptible
Trimethoprim/sulfamethoxazole	≤1/20	Susceptible
Ciprofloxacin	≤0.25	Susceptible
Levofloxacin	≤0.5	Susceptible
Ampicillin	2	Susceptible
Colistin	≤2	Wildtype

Appendix Table 2. Chromosomal virulence factors in *Klebsiella pneumoniae* strains UCLA353, AJ210, SB5881, and 18-0005.

		Alleles			
Virulence factor	Locus	UCLA353	AJ210	SB5881	18–0005
Colibactin	*clbA*	1	1	1	1
	clbB	–	–	4	4
	clbC	1	–	1	1
	clbD	1	–	1	1
	clbE	–	1	1	1
	clbF	1	–	1	1
	clbG	1	1	1	1
	clbH	5	–	5	5
	clbI	1	–	–	1
	clbL	1	–	1	1
	clbM	1	1	1	1
	clbN	1	–	1	1
	clbO	1	1	1	1
	clbP	1	1	1	1
	clbQ	1	1	1	1
	clbR	1	1	1	1
Type 3 fibriae	*mrkA*	2	2	2	2
	mrkA	87	87	87	87
	mrkB	9	–	9	9
	mrkC	10	–	–	–
	mrkC	302	302	302	302
	mrkD	8	8	–	8
	mrkF	2	2	2	2
	mrkI	3	3	3	3
	mrkJ	6	6	6	6
Yersiniabactin	*ybtA*	–	–	1	1
	ybtA	–	39	39	39
	ybtE	1	1	1	1
	ybtP	1	1	1	1
	ybtQ	1	–	1	1
	ybtS	1	7	–	7
	ybtT	–	1	1	1
	ybtU	1	1	1	1
	ybtX	1	–	1	1
	irp1	–	–	16	16
	irp2	–	–	3	3
	fyuA	1	1	1	1
